# Comparison of serum cytokine levels in symptomatic and asymptomatic HIV-*Leishmania* coinfected individuals from a Brazilian visceral leishmaniasis endemic area

**DOI:** 10.1371/journal.pntd.0010542

**Published:** 2022-06-17

**Authors:** Diego Lins Guedes, Elis Dionísio da Silva, Maria Carolina Accioly Brelaz Castro, Walter Lins Barbosa Júnior, Ana Victoria Ibarra-Meneses, Achilleas Tsoumanis, Wim Adriaensen, Johan van Griensven, Valéria Rêgo Alves Pereira, Zulma Maria de Medeiros

**Affiliations:** 1 Department of Parasitology, Aggeu Magalhães Institute–Oswaldo Cruz Foundation (Fiocruz), Recife, Pernambuco, Brazil; 2 Curso de medicina, Núcleo de Ciências da Vida, Centro Acadêmico do Agreste, Universidade Federal de Pernambuco, Caruaru, Pernambuco, Brazil; 3 Department of Immunology, Aggeu Magalhães Institute–Oswaldo Cruz Foundation (Fiocruz), Recife, Pernambuco, Brazil; 4 Parasitology Laboratory, Federal University of Pernambuco, Vitoria de Santo Antão, Pernambuco, Brazil; 5 Département de pathologie et microbiologie. Faculté de médecine vétérinaire. Université de Montréal, Saint-Hyacinthe, QC, Canada; 6 The Research Group on Infectious Diseases in Production Animals (GREMIP), Faculty of Veterinary Medicine, Université de Montréal, Montreal, Canada; 7 Department of Clinical Sciences, Institute of Tropical Medicine, Antwerp, Belgium; Institut Pasteur de Tunis, TUNISIA

## Abstract

**Background:**

Visceral leishmaniasis (VL) remains an important infectious disease worldwide. VL-HIV coinfected individuals can present with atypical clinical forms of VL and have a high risk of VL relapse. Some cytokines have been described as potential markers to diagnose active VL and to predict the severity of the cases. However, few studies have included VL-HIV coinfected patients. We aimed to characterize the levels of several cytokines among VL-HIV coinfected individuals living in a VL-endemic area in Northeast Brazil.

**Methods:**

This was a retrospective, cross-sectional study, aiming to estimate the levels of various cytokines in symptomatic and asymptomatic VL-HIV coinfected individuals. There were 134 study participants (35 symptomatic VL-HIV, 75 asymptomatic VL-HIV, and 24 healthy controls), all ≥ 18 years-old. Serum cytokine levels (interferon-γ, tumor necrosis factor, and interleukins 2, 4, 6, 10, and 17A) were quantified using the Becton Dickinson-BD’s Cytometric Bead Array (CBA) system.

**Results:**

The population mainly consisted of men (64.9%), with a median age of 35 (27–41) years. Asymptomatic individuals were younger (p = 0.013), with more years of education (p < 0.001), and were more often on antiretroviral therapy (p < 0.001) than those in the symptomatic group. Hemoglobin levels (p < 0.001), lymphocytes (p < 0.001) and CD4 count (p < 0.001) were lower in symptomatic individuals, while HIV viral loads were higher (p < 0.001). In the symptomatic VL-HIV coinfected group, we observed increased serum levels of IL-17A, IL-6, and IL-10 compared to asymptomatic patients and the healthy controls. There were no differences in the levels of all cytokines between asymptomatic VL-HIV coinfected individuals and the healthy controls.

**Conclusions:**

Higher serum levels of IL-17A, IL-6, and IL-10 cytokines were observed in symptomatic coinfected individuals but not in asymptomatically infected individuals. More studies among HIV-positive persons are needed to better understand the role of serum cytokines for prognosis, to define cure and predict VL relapses in VL-HIV coinfected individuals.

## Author summary

Visceral leishmaniasis (VL) is a parasitic disease, classified as a neglected disease by the World Health Organization. It is present in more than 60 countries in four continents, with most tropical and subtropical countries affected. Without treatment, the disease is fatal. HIV-positive persons are especially affected by VL, with a worst prognosis. One of the main complications is the frequent reactivation of the disease even after successful treatment (VL relapse). Up to now, it is not clear why and when these reactivations occur, and many researches are trying to find some biological markers to answer this. Cytokines are one of these markers that may explain the progression of the disease. In this study, we compared the level of several key cytokines in symptomatic VL-HIV coinfected patients, asymptomatic VL-HIV coinfected individuals, and healthy controls living in a VL-endemic region in Northeastern Brazil. The serum cytokine levels were higher in symptomatic patients when compared with asymptomatic and healthy controls. More studies following VL-HIV patients are necessary to understand how these cytokines and the other biomarkers vary over time and whether they can predict VL relapse, and also the progression and the prognosis of the disease.

## Introduction

In spite of all the efforts to control visceral leishmaniasis (VL), it remains an important and prevalent infectious disease worldwide. Affecting mainly neglected people in tropical and subtropical nations [1, 2], VL is present in more than 60 countries in four continents [3]. In South America, Brazil carries the highest burden [4]. While most *Leishmania* infections are self-limiting and asymptomatic, symptomatic VL is inevitably lethal without treatment. Despite receiving anti-*Leishmania* drugs and appropriate health care, death is not rare, particularly in HIV coinfected individuals [5]. In addition, VL-HIV coinfected individuals also display higher parasite loads and frequent relapses, compared with VL cases without HIV coinfection [6, 7].

Immunotherapy for VL has been proposed as a potential way to support the treatment with anti-Leishmanial drugs [8, 9]. In fact, despite the disease being known for such a long time, and its considerable lethality [10], only two drugs are available in Brazil–pentavalent antimonials and amphotericin B (in different preparations). These two drugs have significant adverse effects [11], and for some specific patient groups–such as persons living with HIV–the traditional chemotherapy is less effective with higher rates of treatment failure, mortality and relapse [12, 13].

Some cytokines have been described as potential markers for VL. For example, interleukin (IL)-2 could be used for detecting asymptomatic *Leishmania* infection [14–16], IL-6 for predicting the severity of the disease [17–19], interferon (IFN)-γ to define cure after treatment [20, 21], and IL-10 and IFN-γ could be helpful as markers of active VL [22, 23].

However, few studies have included VL-HIV coinfected patients. Consequently, the impact of HIV co-infection on the cytokine profile among patients infected with *Leishmania* is not well understood. In this study, we aimed to compare the levels of several key cytokines between symptomatic and asymptomatic VL-HIV coinfected individuals, and healthy controls living in Pernambuco, a VL-endemic area, in Northeast Brazil.

## Methods

### Ethics statement

All subjects were adults and provided written, informed consent. The study was approved by the research ethics committee of Instituto Aggeu Magalhães, Fiocruz Pernambuco (approval number 51235815.0.0000.5190). The samples were collected partly in 2014 and partly in 2018, and were stored in the freezer (-80°C). At the time of consent, the participants had agreed that the samples could be used by the Instituto Aggeu Magalhães in other future studies.

### Study design and sample

This was a retrospective, cross-sectional study. The main objective of this study was to estimate the levels of different cytokines in symptomatic and asymptomatic VL-HIV coinfected individuals, and healthy controls (negative for VL and HIV). We 1) compared cytokine levels amongst the three groups; 2) assessed the correlation among the levels of cytokines within each group; 3) assessed the correlation between the serum levels of these cytokines and general laboratory data; and 4) examined the association between the CD4 count, HIV viral load and the cytokine levels.

A total of 134 individuals were included (35 symptomatic VL-HIV, 75 asymptomatic VL-HIV, and 24 healthy controls). All patients were at least 18 years-old. Except for the group of healthy controls, all the participants had already been diagnosed with HIV and they were tested for *Leishmania*, presenting at least one positive result on four VL test done: the rK39 rapid test (InBios International, Seattle, USA), and the Direct Agglutination Test (DAT) (Biomedical Research, AD Amsterdam), both done on serum; the KAtex *Leishmania* antigen test on urine (Kalon Biological Ltd, Guildford, UK); and the kDNA Polymerase Chain Reaction (PCR) test on peripheral blood, according to Souza et al [24] and Gualda et al [25]. For the symptomatic group, we used samples of the 35 VL-HIV coinfected patients diagnosed in a previous study in 2014 in three referral hospitals from Recife, Brazil [26]. For the asymptomatic group, we randomly selected samples of asymptomatic VL-HIV cases in 2018 attending an HIV outpatient service in Petrolina, Brazil [27]. All samples were stored in freezer (-80°C). The healthy controls were tested in 2018 for HIV and VL and were found negative for both.

### Data collection and laboratory procedures

The following data were collected from the medical records for the symptomatic and asymptomatic VL-HIV patients: gender, age, education; levels of hemoglobin, leucocytes, neutrophils, lymphocytes, platelets, aspartate aminotransferase (AST), alanine aminotransferase (ALT), urea, creatinine, CD4 and HIV viral load. For the healthy controls, we only collected demographic data and quantified cytokine levels.

Using serum samples, we used the Becton Dickinson-BD’s Cytometric Bead Array (CBA) system to determine the following cytokines: IFN-γ, tumor necrosis factor (TNF), IL-2, IL-4, IL-6, IL-10, and IL-17A, according to the manufacturer’s recommendation. The limits of detection of these cytokines, according to the manufacturer, are: IL-2–2.6 pg/ml; IL-4–4.9 pg/ml; IL-6–2.4 pg/ml; IL-10–4.5 pg/ml; TNF—3.8 pg/ml; IFN-γ - 3.7 pg/ml; IL-17A - 18.9 pg/ml. The flow cytometer FACSCalibur (BD, San Jose, California, USA) was used, with subsequent analysis using the FCAPArray (BD, San Jose, California, USA).

### Statistical analysis

Continuous variables were summarized as medians and interquartile range, while categorical variables were as counts and proportions. Differences in proportions of categorical variables by group were compared using Chi-square or Fisher’s exact test as appropriate. Differences in medians by group were compared using the Mann-Whitney U test. Multiple comparisons of cytokine levels among the three groups were performed using the Dunn’s multiple comparisons test. The Spearman’s correlation coefficient was calculated to assess the correlation between the different cytokines and to assess the correlation between cytokine levels and hematological and biochemical parameters. Data analysis was done with Stata SE 12.0 software (StataCorp, College Station, TX, USA) and GraphPad Prism version 8 (GraphPad Software, San Diego, California, USA).

## Results

### Clinical characteristics

The study population was composed mainly of men (64.9%), with a median age of 35 (27–41) years, and with eight or fewer years of education. Demographic and laboratory characteristics by group are shown in Table 1. Asymptomatic individuals were younger (p = 0.013), with more years of education (p < 0.001), and were more often on antiretroviral therapy (p < 0.001) than those in the symptomatic group. The levels of hemoglobin (p < 0.001), lymphocytes count (p < 0.001) and CD4 count (p < 0.001) were lower in symptomatic individuals, while HIV viral load were higher (p < 0.001) in the latter (Table 2).

**Table 1:** Comparison of demographic characteristics between groups (univariate analysis) from Pernambuco, Brazil.

Characteristic	Symptomatic (N = 35)	Asymptomatic (N = 75)	Healthy controls (N = 24)	p-value
Gender (n (%))				
Male	22 (62.86)	54 (72)	11 (45.83)	0.062
Female	13 (37.14)	21 (28)	13 (54.17)	
Age (years) (median and IQR)	38 (31–48)	33 (26–41)	24 (23–25)	< 0.001
Education (years) (n (%))				
0–8	30 (85.71)	35 (46.67)	0 (-)	< 0.001
> 8	5 (14.29)	40 (53.33)	24 (100)	
On ART (n (%))	22 (62.86)	75 (100)	-	< 0.001

IQR, interquartile range. ART, antiretroviral therapy.

All percentages are column percentages.

**Table 2:** Comparison of laboratory characteristics between symptomatic and asymptomatic VL-HIV coinfected individuals (univariate analysis) from Pernambuco, Brazil.

Characteristic (median (IQR))	Symptomatic (N = 35)	Asymptomatic (N = 75)	p-value
Hemoglobin (g/dL)	10.45 (8.9–11.4)	13.1 (12.2–14.7)	< 0.001
Leucocytes (/mm^3^)	4695 (3030–7360)	5350 (4600–6300)	0.558
Neutrophils (/mm^3^)	2790 (1400–5535)	2576 (2064–3358)	0.348
Lymphocytes (/mm^3^)	880 (538–1300)	1782 (1551–2310)	< 0.001
Platelets x10^3^ (/mm^3^)	227.5 (156–333)	253.5 (207.5–299.5)	0.180
AST (U/L)	31 (20.7–72.4)	25 (21–37)	0.239
ALT (U/L)	30 (20–53.6)	24 (18–38)	0.150
Urea (mg/dL)	27.3 (22.67–39.75)	23 (19–34)	0.065
Creatinine (mg/dL)	0.71 (0.51–0.9)	0.8 (0.7–1)	0.159
LTCD4+ count (cells/μL)	197 (16–414)	587 (422–765)	< 0.001
Characteristic (n (%))			
LTCD4+ count (cells/μL)			
< 200	16 (51.6)	8 (11.6)	< 0.001
200 **-** 349	6 (19.4)	6 (8.7)	
≥ 350	9 (29.0)	55 (79.7)	
HIV viral load (copies/mL)			
Undetectable (< 50)	5 (16.67)	52 (75.36)	
50–100,000	14 (46.67)	15 (21.74)	< 0.001
≥ 100,000	11 (36.67)	2 (2.90)	

IQR, interquartile range; AST, aspartate aminotransferase; ALT, alanine aminotransferase. LTCD4+, lymphocyte T CD4+.

All percentages are column percentages.

### Determination of serum cytokine levels

**Fig 1:**
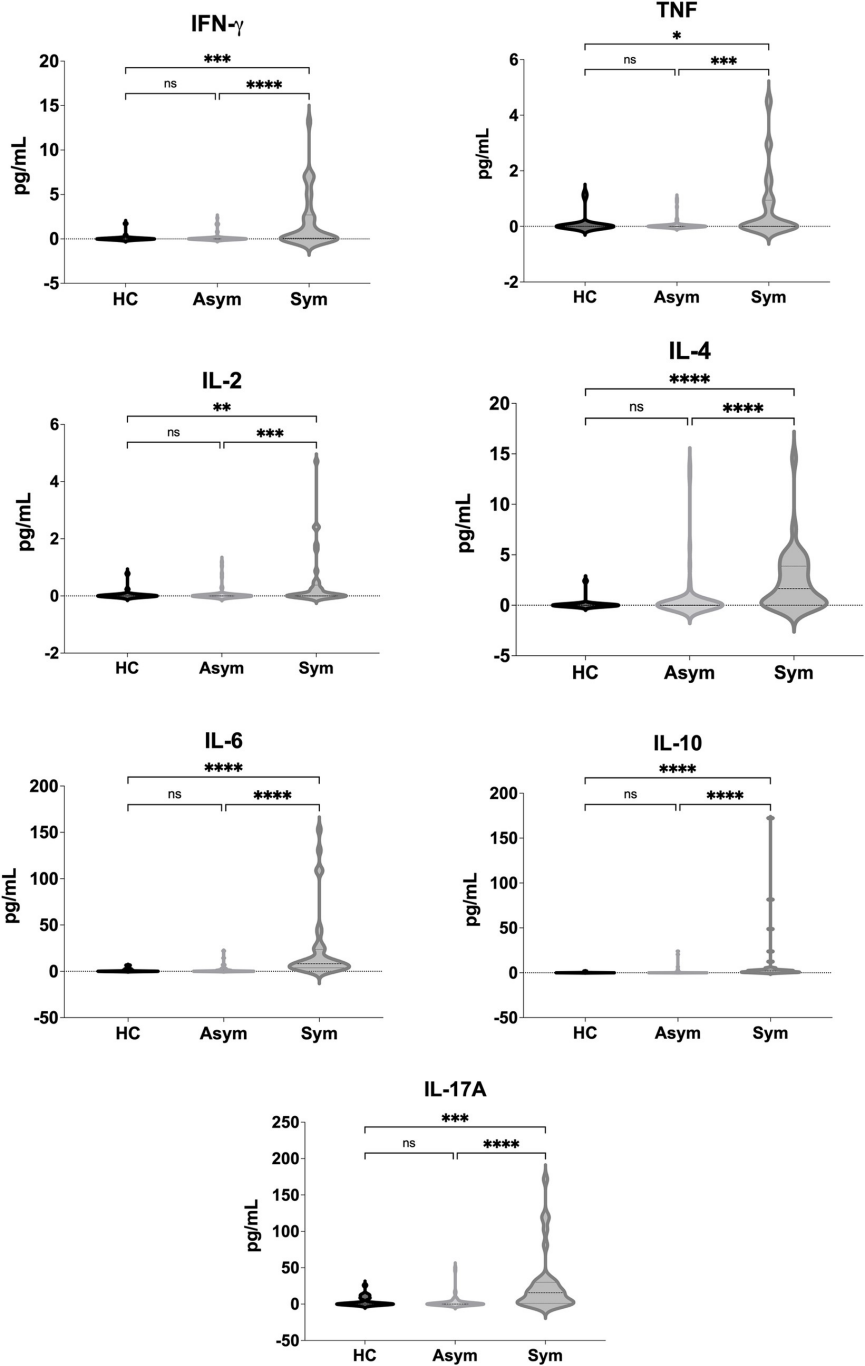
Comparison between serum cytokine levels by disease group (Kruskal-Wallis test with multiple comparisons; median and interquartile range). **ns, not significant;** HC, healthy controls (n = 24); Asym, asymptomatic VL-HIV coinfected patients (n = 75); Sym, symptomatic VL-HIV coinfected patients (n = 35).

**Fig 2:**
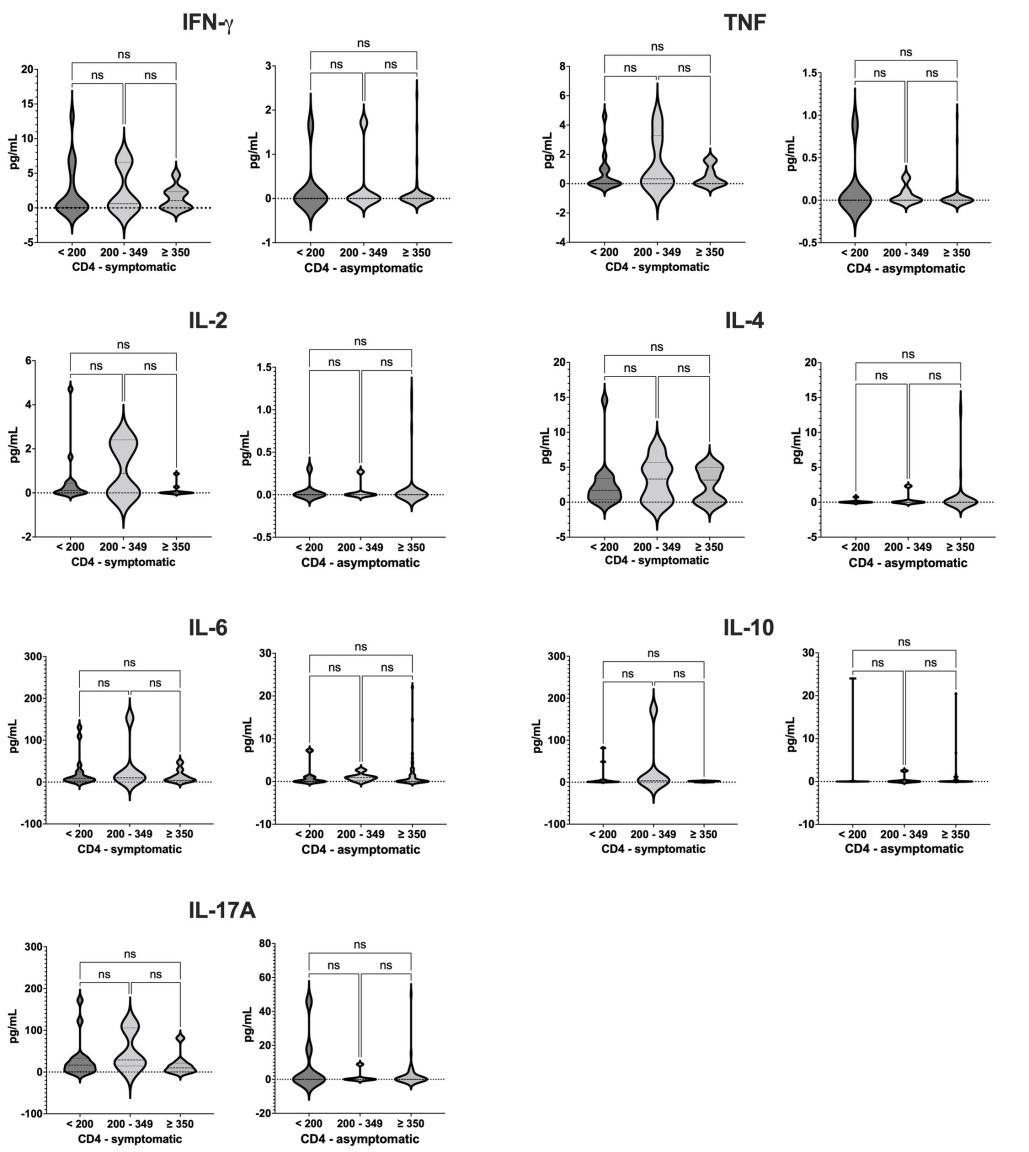
Serum cytokine levels by CD4 count, by disease group (asymptomatic and symptomatic VL-HIV coinfected individuals). ns, not significant.

When compared with the asymptomatic VL-HIV group and with the healthy controls, we observed among the symptomatic VL-HIV coinfected group increased levels of all cytokines tested, mainly of IL-17A, IL-6 and IL-10 (Fig 1). There were no differences in the levels of all cytokines evaluated between asymptomatic VL-HIV coinfected individuals and the healthy controls. We also evaluated whether the lymphocyte T CD4 count was associated with the levels of the various cytokine analyzed. For this, we categorized the CD4 count (< 200, 200–349, and ≥ 350) for each group of coinfected individuals (symptomatic and asymptomatic), and we compared the levels of each cytokine in each CD4 subgroup. We did not observe any statistically significant difference in the analysis (Fig 2).

### Correlations between cytokine levels and laboratory data

The correlations between cytokine levels and hematological and biochemical parameters for the general sample, including CD4 count and HIV viral load, are shown in Table 3. By group, we did not see any statistically significant correlation between cytokines levels and hematological and biochemical parameters among asymptomatic VL-HIV patients. In the symptomatic group, IL-4 was positively correlated with lymphocytes count (rho = 0.5, p = 0.002), and IL-10 was negatively correlated with hemoglobin levels (rho = -0.4, p = 0.020) and positively correlated with AST levels (rho = 0.36, p = 0.049). We did not observe a statistically significant correlation when we compared each cytokine level with the CD4 count and with the HIV viral load in both groups of VL-HIV coinfected individuals.

**Table 3:** Correlation coefficients between serum cytokine levels and general laboratory data of all patients.

	IFN-γ	TNF	IL-2	IL-4	IL-6	IL-10	IL-17A
LTCD4 (cells/μL)	-0.17	-0.17	-0.23 ^a^	-0.22 ^a^	-0.33 ^c^	-0.26 ^b^	-0.28 ^b^
HIV viral load (copies/mL)	0.22 ^a^	0.10	0.24 ^b^	0.34 ^c^	0.40 ^c^	0.39 ^c^	0.28 ^b^
Hemoglobin (g/dL)	-0.35 ^c^	-0.38 ^c^	-0.29 ^b^	-0.35 ^c^	-0.38 ^c^	-0.44 ^c^	-0.39 ^c^
Neutrophils (/mm^3^)	0.04	0.01	0.11	0.14	0.04	0.04	-0.01
Lymphocytes (/mm^3^)	-0.18	-0.17	-0.10	-0.19	-0.42 ^c^	-0.43 ^c^	-0.30 ^c^
Platelets (/mm^3^)	-0.09	-0.13	-0.06	0.04	-0.21 ^a^	-0.22 ^a^	-0.15
AST (U/L)	0.14	0.08	0.14	0.03	0.20	0.26 ^a^	0.25 ^a^
ALT (U/L)	0.04	-0.03	0.09	0.09	0.12	0.19	0.15

IFN-γ, interferon-gamma; TNF, tumor necrosis factor; IL, interleukin; LTCD4, lymphocyte T CD4+.

All values represent the Spearman r.

^a^ *p ≤* 0.05

^b^ *p ≤* 0.005

^c^ *p ≤* 0.0005.

## Discussion

In this study, we measured the serum levels of different cytokines in symptomatic and asymptomatic VL-HIV coinfected individuals living in a VL-endemic area. We also correlated these cytokine levels with laboratorial characteristics. We observed increased levels of IL-17A, IL-6 and IL-10 in the symptomatic VL-HIV coinfected group. In this same group, low levels of IL-2, IFN-γ and TNF were observed. Low levels of all cytokines analyzed were found in samples from asymptomatic VL-HIV coinfected individuals and from the healthy controls.

Usually, it is expected a predominantly T helper type 2 (mainly IL-4, IL-6, and IL-10) response in symptomatic *Leishmania* infections [18, 28], however, a mixed T helper type 1 (mainly IL-2, IFN-γ)/T helper type 2 response could also be seen [29, 30]. Probably, this dichotomy is not the only factor involved in the course of infection [28, 31], and other variables such as parasitic load, nutritional status, and genetic factors, might have an important role in the development and outcome of the disease [32, 33].

In our study, IL-6 levels were sixteen times greater in symptomatic individuals when compared with the asymptomatic individuals and with the healthy controls. High levels of IL-6 have already been observed in patients with active VL, and this could be related to more severe cases and death [18, 19]. Increased IL-6 levels are also related to HIV replication [34, 35], which could explain the high levels in our group of symptomatic VL-HIV coinfected patients, as they presented more frequently with detectable HIV viral load. Similarly, a negative correlation between IL-6 and CD4 count was previously reported [34], with high CD4 counts associated with low IL-6 levels. This could have been reflected in the low values observed in the healthy controls and in the asymptomatic VL-HIV coinfected individuals–the last ones presenting high LTCD4+ levels (median of 587 cells/μL) when compared with those who were symptomatic (median of 197 cells/μL).

It is known that IL-6 influences IL-17 production [36], and this could explain why these two cytokines were the highest expressed. IL-17, an interleukin related to the Th17 profile, was described having a protective effect against the *Leishmania (L) donovani* infection at high levels [37]. In our study, IL-17A presented the highest levels, particularly among the symptomatic patients. This could be due to the fact that the Th17 response is necessary to control infections caused by intracellular pathogens [36]. Higher serum levels of IL-17A have been described in patients with active VL [38]. IL-17A levels started to decrease during the treatment, but remained higher compared with uninfected study participants [39]. Furthermore, IL-17 has been associated with susceptibility to *L*. *donovani* infection in an animal model [40], which likewise might explicate our findings.

Regarding IL-10, we detected levels twelve times higher in symptomatic individuals when compared with asymptomatic individuals and with the healthy controls. In the asymptomatic group, we observed low serum concentrations of IL-10, not significantly higher than in the healthy controls. It was previously demonstrated that IL-10 levels are higher during active VL [14, 18, 30]. Further, some studies suggest an association between high IL-10 levels and the severity of the disease [17, 23]. The opposite is also true, where asymptomatic individuals often present low levels of IL-10 [41]. High levels of IL-10 could inhibit the human immune response against *Leishmania* parasites [31]. In addition, IL-10 has a regulatory role on the Th1 immune response, which in turn, when exacerbated, presents a harmful effect on the host. Therefore, the balance of IL-10 production may be determinant to the progression of the disease. We also observed in symptomatic individuals a positive correlation between IL-10 and AST levels, which have previously been found to be associated to active VL [18, 42]. Active VL could also explain the negative correlation we observed between IL-10 and hemoglobin levels, since anemia is a frequent finding in patients with VL.

In this study we used stored serum samples, which could be seen as a potential limitation since the levels of the cytokines might decrease overtime and could be affected by a freeze-thaw cycle. As a cross-sectional study, we cannot infer about the clinical outcomes of the participants and their correlation with the various cytokine levels. Moreover, since we did not explore the presence of other opportunistic infections, their role on the cytokine levels of the patients evaluated could act as a confounder factor. Further cohort studies could clarify the potential influence of other opportunistic and latent coinfections (e.g., toxoplasmosis or tuberculosis) on the immune profile of the VL-HIV coinfected cases.

## Conclusions

We observed among the symptomatic VL-HIV coinfected participants high levels of IL-17A, IL-6 and IL-10, compared with the asymptomatic coinfected individuals and the healthy controls. The LTCD4+ count showed no relation with the cytokine levels. The cytokines IL-6 and IL-10 seems to be related with active VL even in cases of coinfection with HIV. More studies among HIV-positive patients are needed to better understand the role of these cytokines in terms of prognosis, prediction of cure or relapses in these coinfected individuals. Such information could better guide prophylaxis and treatment–and consequently might reduce lethality and unnecessary medical interventions. Cohort studies, and studies based on cell stimulation with *Leishmania* antigens, and with a more wide-ranging group of cytokines and other biomarkers, can be useful to fill these gaps.
